# Progress in Genomic Mating in Domestic Animals

**DOI:** 10.3390/ani12182306

**Published:** 2022-09-06

**Authors:** Pengfei Zhang, Xiaotian Qiu, Lixian Wang, Fuping Zhao

**Affiliations:** 1 Institute of Animal Science, Chinese Academy of Agricultural Sciences, Beijing 100193, China; 2 National Animal Husbandry Service, Beijing 100125, China

**Keywords:** genomic mating, domestic animals, inbreeding

## Abstract

**Simple Summary:**

Since animal domestication, breeders have been selecting candidates for breeding based on phenotypic performance. Estimating breeding values through the best linear unbiased prediction method represents a revolutionary shift in animal breeding. On this basis, selection and mating are utilized to improve the production level of animals. The application of genomic selection has once again revolutionized animal breeding methods. However, although this kind of truncated selection based on breeding values can significantly improve genetic gain, the genetic relationship between individuals with a high breeding value is usually closed, and the probability of being co-selected is greater, which will lead to a rapid increase in the rate of inbreeding in the population. Reduced genetic variation is not conducive to long-term sustainable breeding, so a trade-off between genetic gain and inbreeding is required. Genomic mating is the use of candidate individuals’ genomic information to implement optimized breeding and mating, which can effectively control the rate of inbreeding in the population and achieve long-term and sustainable genetic gain. It is more suitable for modern animal breeding, especially for conservation and genetic improvement of local domestic animal breeds.

**Abstract:**

Selection is a continuous process that can influence the distribution of target traits in a population. From the perspective of breeding, elite individuals are selected for breeding, which is called truncated selection. With the introduction and application of the best linear unbiased prediction (BLUP) method, breeders began to use pedigree-based estimated breeding values (EBV) to select candidates for the genetic improvement of complex traits. Although truncated selection based on EBV can significantly improve the genetic progress, the genetic relationships between individuals with a high breeding value are usually closed, and the probability of being co-selected is greater, which will lead to a rapid increase in the level of inbreeding in the population. Reduced genetic variation is not conducive to long-term sustainable breeding, so a trade-off between genetic progress and inbreeding is required. As livestock and poultry breeding enters the genomic era, using genomic information to obtain optimal mating plans has formally been proposed by Akdemir et al., a method called genomic mating (GM). GM is more accurate and reliable than using pedigree information. Moreover, it can effectively control the inbreeding level of the population and achieve long-term and sustainable genetic gain. Hence, GM is more suitable for modern animal breeding, especially for local livestock and poultry breed conservation and genetic improvement. This review mainly summarized the principle of genomic mating, the methodology and usage of genomic mating, and the progress of its application in livestock and poultry.

## 1. Introduction

Selection and mating are two important components of domestic animal breeding programs. The goal of such programs is to maximize the genetic gain, while restricting the rate of inbreeding in the nucleus breeding population. Selection is used to screen out superior individuals with higher breeding values for breeding in the next generation, which can lead to an increase in the frequencies of favored genes in the population. Mating is used to consciously set an optimal mating plan that realizes a combination of favored genes to produce elite offspring. Therefore, selection is the premise of mating, and both are complementary; selection and mating can improve the genetic performance of livestock and poultry.

Recently, domestic animal breeding has entered the genomic era. Numerous genomic data have been generated, which provides a new path to estimating the breeding value and inbreeding coefficient of an individual. Using genomic data, the realized relationship of selected candidates can be directly calculated. Traditionally, the relationships are computed from pedigree information, which comprises the statistical expectation of the probability of identity by descent (IBD) [1,2], and its accuracy strongly depends on the completeness and accuracy of pedigree data [3]. Thus, genomic estimated breeding values (GEBVs) based on genomic information are more accurate than EBVs based on pedigree information. The application of genomic selection (GS) in livestock and poultry gave rise to an unprecedented acceleration in genetic progress, particularly in dairy cattle populations [4]. However, incorporating GS in breeding programs could potentially lead to an increased rate of inbreeding. Although the inbreeding rate of GS per generation is lower than pedigree selection, GS could lead to higher inbreeding rates per year when compared to conventional breeding schemes [5]. Inbreeding not only leads to inbreeding depression but also decreases genetic variance, which can reduce genetic gain in the long term.

The question of how to effectively balance increasing genetic gain and controlling the rate of inbreeding in the nucleus breeding population, while maintaining genetic diversity is always important in animal breeding. In the late 20th century, numerous algorithms to implement optimal mating schemes were proposed on the basis of pedigree relationships [6,7,8]. These methods seek to not only maximize genetic gain but also minimize progeny inbreeding and maintain genetic diversity. Currently, genomic information is integrated to determine which genotypes should be combined to produce the next breeding population, which is also called genomic mating (GM) [7]. Similar to GS, in GM, it is necessary to compute the genetic relationship between selected candidates and estimate marker effects. In addition, GM can integrate the genomic information and the estimated marker effects to determine which genotypes should be kept to produce the offspring. Moreover, we can obtain information on recessive carrier status or major gene segregation in the population. Therefore, GM can use genomic information to obtain the optimal mate allocates, which is more accurate and reliable than using pedigree information [3]. Using genomic information, genomic mating can improve the expected genetic values of offspring, maintain genetic diversity, and reduce the frequencies of lethal recessive alleles.

In this review, we provide a broad overview of the developments in the basic theories, methodology, and usage of genomic mating. Moreover, the current status of application of genomic mating in livestock and poultry are summarized. 

## 2. Concept of Genomic Mating

In 2016, genomic mating was proposed by Akdemir et al. [7]. It is an optimized genetic improvement method for candidate selection and mating using genomic information to track the transmission of parental genetic material, and more accurately estimate Mendelian sampling and the actual genetic contribution of parents to their offspring. Compared to traditional mating, genomic mating no longer relied on pedigree data. Moreover, it can fully utilize the genomic information of candidate individuals because non-additive effects are also taken into account. However, at present, the estimation of genomic breeding value and the actual selection schemes are mainly focused on the additive effect that can be stably inherited, while the dominant effect that can be reflected by specific allele pairing has not been exploited in realistic scenarios of genomic selection. The SNP chip data can provide all individuals’ genotypes, which can be used to produce superior individuals with ideal genotypes after optimal mate allocation. Moreover, genomic mating fits the inbreeding coefficient as a regression term that can be used not only to eliminate the influence of inbreeding on the past but also to predict its future performance.

In terms of whether there is human intervention, mating plans can be classified into random and non-random mating [9]. On the basis of the relatedness with the mating pairs, they can be divided into inbreeding and non-inbreeding [9]. According to the correlation of mating pairs’ genotypes and/or phenotypes, they can be separated into positive assortative mating and negative assortative mating [10]. Positive assortative mating indicates a tendency to mate with genetically or phenotypically similar individuals, while negative assortative mating suggests a tendency to mate with genotypically or phenotypically dissimilar individuals [11].

## 3. Usage of Genomic Mating

### 3.1. Inbreeding Control

Controlling inbreeding is one of the most important aspects of mating programs. Traditionally, the expected inbreeding of offspring from a given mating scheme is calculated based on pedigree relationships between parents [12]. However, the depth of available pedigrees, coupled with known kinship errors [13], influences the effectiveness of this approach. Furthermore, pedigree methods cannot distinguish individuals with the same pedigree that have inherited different parts of the genome and therefore have different realization relationships. It has been suggested that GEBVs can also be used as a tool to reduce inbreeding, as they can explain more Mendelian sampling variation than BLUP [1]. Therefore, selection for GEBV is expected to increase genetic gain, while maintaining population diversity. However, what proportion of the Mendelian sampling variance is explained by GEBV and whether GEBV can be used to manage inbreeding still remain unclear. Genomic mating can effectively control inbreeding, even in commercial populations with incomplete or no pedigrees. Genomic mating uses genomic information to provide a more accurate estimate of the relationship coefficients between parents and, in doing so, a more accurate estimate of the expected inbreeding of offspring [14]. Pryce et al. [3] investigated effects of the breeding schemes using pedigree, genomic, and ROH information to control the degree of inbreeding by analyzing the expected genetic gain of the mating progeny and the changes in inbreeding and homozygosity for recessive deleterious alleles. The results indicated that the use of genomic information in the breeding program was a more effective method to reduce the expected inbreeding coefficient of offspring than using pedigree information, with minimal impact on genetic progression. Using genomic information could reduce the expected degree of inbreeding of progenies compared to using pedigree information given the same genetic gain. Moreover, genomic mating can measure the rate of inbreeding in specific genomic regions [15], sex chromosomes, and mitochondrial DNA [16] so as to minimize their loss of diversity.

### 3.2. Breed Conservation

The goals of breed conservation or breeding programs are affected by many factors, which include economic value, historical bottlenecks, and maintenance of genetic diversity [8]. Breeding values of target traits are used to improve the economic value of most livestock and poultry breeds. Therefore, the most crucial breeding goal is to maximize genetic gain [17]. However, domestic animals [18] often experienced historical bottlenecks due to the overuse of elite males. Under this condition, it is imperative to minimize the rates of inbreeding in these animal populations. To maximize genetic gain, people prefer to select animals with the highest breeding values of economically important traits, which will increase the rates of inbreeding and may lead to severe inbreeding depression and new bottlenecks. On the other hand, only focusing on maintaining genetic diversity is not conducive to the economics of conservation farms.

However, current conservation methods do not combine the conservation of genetic resources with the genetic improvement of productive performance. In protected domestic animal breeds, it is also very important to select their specific traits, which will help to further consolidate their dominance and maintain their uniqueness. From a long-term sustainability perspective, the issue of how to combine conservation with selective breeding in the conservation field is crucial. While maintaining the overall genetic diversity of local domestic breeds, great attention should be paid to the genetic improvement of economically important traits to adapt to the sustainable development. The current study demonstrated that genomic mating was an effective method for balancing inbreeding and genetic gain, maintaining high genetic diversity across the genome. Zhao et al. [19] showed that using optimal contribution selection based on genomic information (GOCS) not only achieved higher genetic gain but also maintained a relatively high level of genetic diversity. Furthermore, as the number of generations increased, the advantages of GOCS become more obvious in maintaining genetic diversity. The results suggested that GOCS was a better option if one wanted to combine conservation and genetic progress in practical production on conservation farms. Sanchez-Molano et al. [20] reported that a genome-based optimal contribution strategy could effectively control the rate of increase in inbreeding, while guaranteeing genetic improvement in target traits.

### 3.3. Heterozygous Advantage

In multi-breed livestock populations, the magnitudes of dominance effects differed between and within breeds for many economically important traits. Dominant effects are not inherited from a single animal but through pairs of animals because these effects are caused by the interaction of pairs of genes at the same locus, and only one gene of each pair is passed on to individual offspring [21]. The dominant effect is important, especially in the field of genomic mating and cross-breeding. In livestock and poultry populations, dominant effects are not widely exploited. This is because pedigree-based relationships of individuals do not provide enough information, since accurate EBVs or GBVs usually require large full-sibling families. In highly productive animals, such as chickens and pigs, full siblings and half siblings are highly mixed. In addition, dominant effects are rarely included in models in routine genetic evaluation [22]. Using SNP information, we can directly observe the genotypes of the heterozygosity [23]. With the increasing number of genotyped individuals, incorporation of dominant effects into genetic evaluation models is also an option. Incorporating dominant effects into genetic evaluation models can improve prediction accuracy. 

However, selecting parents based on additive merit only before mate assignment may preclude the selection of parents that, when used in particular mating, would produce offspring with high overall genetic values, which include additive effects and dominance effects [24]. By using genomic mating, different gene combinations that may exist between loci can be mined to explore better breeding patterns. By exploiting the dominant effect, hybrid breeding can be realized and the heterosis can be further utilized. When dominant effects are included in the breeding model, the predictive model can more accurately predict the possible effects of breeding. Dominant effects can be used in terminal hybrid systems to select purebred animals by integrating dominant effects to maximize their hybrid performance [25,26].

## 4. Methodology of Genomic Mating

### 4.1. Linear Programming

Linear programming was one of the earliest methods used for optimal mate allocation [27]. Linear programming is an optimization pairing method that is constrained by linear equations or linear inequalities, calculates the expected offspring values for all possible mate pairs, and optimally solves the objective function. Linear programming obtains the less correlated pairs for simultaneous rather than sequential mating [28]. Usually, in the realistic mating scheme, the maximum usage times of each breeding sire are limited due to its physiological limits. Therefore, the linear programming objective function is usually defined as below:

(1) $$f_{optim}\left(\hat{g_{ij}}\right)=\underset{i=1}{\sum }\underset{j=1}{\sum }\hat{g_{ij}}\;x_{ij}$$

where *g*_ij_ is the expected genetic value of the progeny of male *i* and female *j*; *x*_ij_ is a binary decision variable. When *x*_ij_ is 0, it means that this mate pair is not adopted, and when *x*_ij_ is 1, it means this pairing will be kept. Usually, the number of uses of male and female animals is limited: for each male animal *i*, *x_i_*_1_ + *x_i_*_2_ +...+ *x_ij_* ≤ *n* means that, for each male animal *i*, the maximum number of uses is *n*; *x_i_*_1_ + *x_i_*_2_ +...+ *x_ij_* =1, which means that, for each female *j*, there needs to be one and only one breeding male paired with it.

### 4.2. Genomic Optimal Contribution Selection

In 1994, Woolliams et al. [29] proposed the theory of genetic contribution, which holds that during the actual Mendelian sampling of parental individuals and their genetic contributions to the offspring population, there was a certain threshold linear relationship, and the genetic gain could be maximized under the premise of constraining the relationship between parents. In 1997, Meuwissen et al. [6,30] proposed the optimal contribution selection (OCS) method. This method was based on the pedigree information of the animals selected for breeding, by maximizing the weighted genetic value of the parents, while limiting the relationship between each other, thereby providing sustainable, long-term genetic gain for genetic selection [31,32,33]. Let *A* be the pedigree additive genetic relationship matrix, and *c* be the vector of the proportion of the genetic contribution of individuals to the next generation in the random mating scheme. Given the value of the *c* vector, the average additive genetic correlation can be calculated as *r* = (1/2) *c′Ac*. Let *b* be the EBV vector of the backup candidate individuals (i.e., the EBV estimated by BLUP); then, the expected genetic gain of the next generation (i.e., the average EBV of the offspring) is *β* = *c’b*. If the breeding goal is to obtain the expected genetic gain while keeping the genetic relationship increments within a minimum, the mating problem can be expressed as:

(2) $$\begin{array}{ll} \underset{c}{minimize} & r=\frac{1}{2}c^{’}Ac\\ subject\;to & c^{’}b=\rho \\ & c^{’}1=1\\ & c\geq 0 \end{array}$$

Sonesson et al. [34] replaced the pedigree-based additive genetic relationship matrix (*A*) with the genomic genetic relationship matrix (*G*) in the OCS method. The EBV estimated vector *b* is also replaced by the GEBV vector *g*, and the genomic mating can be implemented as follows:

(3) $$\begin{array}{ll} \underset{c}{minimize} & r=\frac{1}{2}c^{’}Gc\\ subject\;to & c^{’}b=\rho \\ & c^{’}1=1\\ & c\geq 0 \end{array}$$

In theory, a pedigree-based *A* matrix is the expected value of true genetic relationships. The additive genetic relationship calculated based on pedigree differs from the actual relatedness due to Mendelian sampling [35]. *G* matrix is the realized genetic relationship, which reflects the actual Mendelian sampling and is, therefore, more accurate. Moreover, the use of the *G* matrix avoids problems, such as pedigree errors or absence, as well as ignoring biases caused by older generations when computing the A matrix [3]. In addition, using *A* matrix in OCS seems to target autosomal genes, probably with (almost) equal weighting to all genes, while *G* matrix can take account of variation in the level of relationship not only between animals within the same family but also between genomic regions [15], sex chromosomes, and mitochondrial DNA [16].

### 4.3. Genetic Algorithm

Genetic algorithms are particularly suitable for obtaining the optimal solution of combinatorial problems [7]. The optimal mating combination required to obtain the next breeding population is determined according to the effective frontier surface (EFS). The EFS method allows breeders to understand how the expected risk of a breeding program varies with the inbreeding coefficient. The optimized breeding scheme can increase the genetic variance within the population, slow down the increase of inbreeding coefficient and kinship coefficient, and in the meantime ensure a certain genetic progress. The formulation of the mating problem can consolidate the measures of inbreeding and risk as follows:

(4) $$\underset{P}{minimize}r(\lambda_{1},\lambda_{2},p)=-risk(\lambda_{1},p)+\lambda_{2}\times Inbreeding(p)$$

where λ_1_ and λ_2_ are the tuning regulator, and *P* is the mating matrix. λ_1_ controls allele heterozygosity weighted by the marker effects, λ_2_ is the parameter whose magnitude regulated the amount of co-ancestry in the progeny. In this sense, the efficient mating problem can be stated as an optimization problem:

(5) $$\begin{array}{ll} \underset{P_{}}{minimize} & Inbreeding(p)=1{’}_{N_{c}}(P_{}GP_{}^{’}+D_{3})1_{N_{c}}\\ subject\;to & \;\;\;Risk(P_{},\lambda_{1})=\rho \end{array}$$

where *D* is the diagonal variance of founder effects, and *N*_c_ is the number of offspring.

Genomic mating focuses on mate allocation rather than truncated selection. Although both GM and GS need estimate marker effects, in GM, genetic information and estimated marker effects are used to decide which kinds of genotypes should be crossed to obtain the optimal genotypic combination in the following generation. The biggest difference between genomic mating and genomic selection is that the former directly selects the optimal mating pairs from the candidates, instead of truncated selection, which is based on breeding values. Furthermore, genomic mating takes account of the complementary information of the mating parents. Therefore, genomic mating uses genomic information more comprehensively than genomic selection. Akdemir et al. [7] showed that GM is more suitable for improving complex traits than phenotypic selection and GS by simulation.

### 4.4. Other Methods

Minimum co-ancestry and minimizing the covariance between ancestral contributions (MCAC) have also been proposed based on long-term genetic contribution theory. Minimum-co-ancestry mating has long been recommended in practical breeding schemes because it minimizes the variation of each ancestor’s contribution across allocated mating [36,37]. This method could exploit genomic information by maximizing the probability that all ancestors contribute chromosomal segments to all allocated matings. In comparison to minimum-co-ancestry mating, MCAC mating is a new design that may be particularly responsive to genomic information by maximizing the number of combinations of chromosomal segments from ancestral animals in allocated mating. They disperse the contributions within breeding populations and increase the number of ancestors that contribute to each descendent [2,38,39]. This enables selection to bring ancestry closer to the exact threshold linear relationship and reduces inbreeding rates. Both MC and MCAC mating can be extended to include genomic information by replacing their pedigree relationship matrices with single or multiple relationship matrices proposed to control for parental relationships. Thus, genomic relationships can be used to develop mating designs that more appropriately spread genetic contributions across breeding populations, thereby increasing long-term genetic gains.

Kinghorn published a series of papers [40,41,42] about the use of a general heuristic algorithm to separate optimization and objective function into mate selection methods and transformed the problem of calculating the contribution rate of candidate individuals into selecting optimal mate allocation. The mate selection in these articles involves two components: (i) the mate selection index (MSI), which includes constraints on genetic gain, genetic diversity, use of offspring inbreeding and reproductive technologies, genotype frequencies of key markers, and resource constraints; and (ii) the mate selection algorithm used to find mating sets that maximize MSI. These strategies were named look-ahead mate selection (LAMS) schemes because they involved predicting mate selection in offspring [43], and they considered within-cross variance [44].

## 5. Current Status of the Implementation of Genomic Mating in Livestock and Poultry

### 5.1. GM in Cattle

Clark et al. [45] investigated the effects of using genomic information to find the optimal contribution selection based on simulated and real data on dairy bulls. Using genomics to estimate the breeding values can increase the genetic gain for optimal contribution selection. GEBVs were more accurate and showed more intrafamilial variation, which led to higher genetic gain under the same restrictions on inbreeding. Carthy et al. [46] compared the effects of three mating methods using genomic information (random mating, sequential selection, and linear programming) on the mating plan of cattle based on bovine simulation data. The results demonstrated that linear programming methods using genomic relationships and mating assignments within the proposed exponential framework reduce not only the expected level of genomic inbreeding in the herd but also the variability between and within offspring of the genetic value of the selected traits. Due to increasing inbreeding, the accumulation of recessive genes and the risk of inbreeding decline. Holstein dairy breeding programs should consider aspects that both maximize genetic gain and minimize long-term genetic relationships. Schierenbeck et al. [47] developed the use of semidefinite programming with pedigree-based and genomic relationships to control inbreeding and maximize genetic progress, which, compared to previous applications using GENCONT software [48] and pedigree-based relationships, focused on semidefinite programming (SDP) and relationships constructed from SNP data. For traits with medium and low heritability, the method can identify those matings to ensure maximum genetic gain within the specific constraints of the largest relationship. Aliloo et al. [49] evaluated effects on different mating programs using genomic data of dairy cows. Results showed that mating programs including dominance and heterozygosity effects outperformed a model with only additive effects, and genomic mating programs with non-additive genetic effects increased milk, fat, and protein yields by up to 38, 1.57, and 1.21 kg, respectively. Compared with random mating, the addition of the dominance effect and heterozygous effect shortened the calving interval by 0.70 d. Compared with random mating, the mean reduction in inbreeding of offspring with non-proliferative genetic effects added to mating was 0.25–1.57 and 0.64–1.57 percentage points of calving interval and production traits, respectively. The reduction in inbreeding was accompanied by an increase in average profit per mating of AUD 8.42 for the model with additive, dominance, and heterozygous effects compared to random mating. However, mate allocation that benefits from non-additive genetic effects improves offspring performance only in the generation where it is implemented, and gains in specific combinatorial abilities do not accumulate across generations. Continuous updating of genome prediction and mate assignment programs is required to benefit from non-adaptive genetic effects in the long term. Bengtsson et al. [50] employed linear programming to obtain the optimal mate scheme for Nordic red cows integrating genomic information. The mating results suggested that it was possible to reduce differential genetic relationships between parents with minimal impact on the genetic level. Including known recessive genetic defects eliminates the cost of expressing genetic defects. Using genomic information can better control the inbreeding level between mating individuals. Linear programming maximizes economic scores for all study herds in seconds, meaning it is suitable for implementation in mating software for use by advisors and farmers. Bérodier et al. [51] compared three mate assignment strategies (random mating, sequential mating, and linear programming mating) on 160 actual Montbéliarde populations using male and female genomic information, and the ratio of genomic information. They used pedigree information to more efficiently maximize genetic gain, while limiting expected inbreeding of offspring and the risk of breeding offspring homozygous for lethal recessive alleles. The findings also show that the linear programming algorithm was proven to be an attractive method for mate allocation because it is fast, while taking into account all herd-specific constraints to maximize the expected return to the farmer.

### 5.2. GM in Pigs

He et al. [52] performed genomic optimum contribution selection (GOCS) versus no-control inbreeding genomic selection in Ningxiang pigs, which are one of the local pig breeds in Hunan province, China. The results showed that each generation of the genetic gain obtained using GS was greater than the strategy of GOCS in the first few generations, but the difference between GS and GOCS decreased rapidly in later generations. Reduction in genetic variation in GS and fixation of causative genes resulted in lower genetic progression than in GOCS. The rate of inbreeding in GOCS mostly remained below 5% per generation, while it increased to 10.5–15.3% per generation with GS. These findings indicated that GOCS is a sustainable strategy for genetic improvement of local breeds. Zhao et al. [19] compared the long-term effects of conventional conservation and optimal contribution selection methods on genetic diversity and genetic gains in local pigs in China based on simulated data. The results showed that optimal contribution selection methods based on pedigree (POCS) or genome (GOCS) information showed more genetic gain than conventional methods, with POCS achieving the greatest genetic gain. However, the GOCS approach can not only achieve higher genetic gain but also maintain relatively high levels of genetic diversity. Studies have shown that GOCS is a better choice for combining conservation and breeding in pigs. González-Diéguez et al. [53] used a linear programming approach to account for non-additive genetic effects in SNP-based mate allocation. The results showed that genomic mate allocation improved the performance of future offspring of −0.79 days, −0.04 mm, and 11.3 g for age at 100 kg, backfat depth at 140 days, and average piglet weight at birth within the litter, respectively.

### 5.3. GM in Other Livestock and Poultry

Raoul et al. [54] developed a deterministic model and used a sequential quadratic programming approach to determine the optimal mating strategy in terms of the economics of major genetic heterozygous carriers in a purebred lamb breeding program, identifying the optimal combination of maximizing profitability at the level of population. Fernández et al. [55] using simulated data to investigate the efficiency of using genomic information in conservation programs and found that the effective population size (*N*e) was significantly increased when calculating the minimum-co-ancestry matings conditioned on markers, but the observed early levels of genetic diversity (either genetic or allelic diversity) were comparable to the diversity obtained with pedigrees alone. Toro et al. [56], using simulated data, also showed that mate allocation provided up to 22% additional selection response in expected offspring compared to random mating. Liu et al. [57] compared the ΔF and ΔG achieved by the minimum-co-ancestry mating (MC) and minimizing the covariance between ancestral genetic contribution (MCAC) strategies with pedigree and genomic information across five breeding schemes using stochastic simulations, also simulating random mating as a reference point. The results showed that MCs and MCACs with genomic information had ΔFs that were 6% to 22% lower than MCs and MCACs with pedigree information without affecting the ΔG of the breeding scheme. MC and MCAC achieved similar ΔF and ΔG. MC and MCAC with genomic information achieved 28% to 44% ΔF, respectively, and up to 14% more ΔG than RAND. These results suggest that MCs and MCACs with genomic information are more effective than those with pedigree information in controlling inbreeding rates. This means that genomic information should not only be used to predict breeding values and cut-off selections in breeding schemes.

## 6. Conclusions

Genomic mating took account of more factors than previous approaches to achieve optimal selection and mating of breeding animals. Compared with genomic selection, genomic mating can consider complementary relationships between mating pairs, as well as some breed characteristics, reduce the level of population inbreeding, and alter population gene frequencies. Current research on genomic mating is mainly aimed at the additive genetic effect of a single trait. At present, most GM studies are still computer simulation studies, and not widely used in practical animal production. Therefore, the issue of how to effectively apply the GM method to the genetic improvement of domestic animals still needs further exploration. Briefly, genomic mating is still at the preliminary research stage, and many problems remain to be addressed, such as the optimization algorithm of GM. The high-throughput computing of GM is also an issue, especially for multi-trait systems that include non-additive genetic effects.

## Data Availability

Not applicable.
